# Endocannabinoid system attenuates emotional stress-induced orofacial musculoskeletal pain in rats

**DOI:** 10.1590/1414-431X2026e15331

**Published:** 2026-08-07

**Authors:** S.S. Silveira, B.W.F. Alves, M.R.P. Lisboa, J.J.R. Nogueira, K.L.S. de Menezes, A.F. Pereira, R.F. Lima, D.V. Gondim, M.L. Vale

**Affiliations:** 1 Programa de Pós-Graduação em Farmacologia, Departamento de Fisiologia e Farmacologia, Faculdade de Medicina, Universidade Federal do Ceará, Fortaleza, CE, Brasil; 2 Programa de Pós-Graduação em Ciências Morfofuncionais, Departamento de Morfologia, Faculdade de Medicina, Universidade Federal do Ceará, Fortaleza, CE, Brasil; 3 Programa de Pós-Graduação em Odontologia, Departamento de Odontologia Clínica, Universidade Federal do Ceará, Fortaleza, CE, Brasil; 4 Centro de Pesquisa e Desenvolvimento de Medicamentos, Faculdade de Medicina, Universidade Federal do Ceará, Fortaleza, CE, Brasil

**Keywords:** Facial pain, Myofascial pain syndromes, Psychological distress, Endocannabinoids, Cannabinoid receptor CB_1_, Cannabinoid receptor CB_2_

## Abstract

Orofacial musculoskeletal pain (OMP) is a common feature of temporomandibular disorders (TMD), a group of conditions affecting the temporomandibular joint, chewing muscles, and associated structures. The etiology of muscular TMD is currently understood within a biopsychosocial model of pain, highlighting the complexity related to OMP. In this context, the objective of this study was to investigate the OMP induced by psychological/emotional stress (ES) in rats, regarding the role of the endocannabinoid system (ECS) through CB_1_ and CB_2_ receptors. Male Wistar rats were divided into Control and ES groups. OMP was induced by ES using the communication box model and evaluated through the mechanical threshold in masticatory muscles. c-Fos, CB_1_, and CB_2_ immunostaining was evaluated in the trigeminal ganglion (TG) and in the trigeminal nucleus caudalis (Sp5C). The selective CB_1_ or CB_2_ antagonist (AM251 or AM630, respectively) or the cannabinoid receptor non-selective agonist (WIN55,212-2) were administered to both groups, and the OMP was evaluated. The mechanical sensitivity of the masticatory muscles increased in the ES group, accompanied by increased c-Fos expression in the TG and Sp5C. AM251 and AM630 increased mechanical sensitivity, while WIN55,212-2 decreased the OMP. Furthermore, ES increased CB_1_ density in the TG and elevated both CB_1_ and CB_2_ in the Sp5C. Additionally, CB_2_ was increased in the masseter muscle. Thus, cannabinoid receptors played a protector role in OMP caused by ES, indicating that cannabinoid drugs or the modulation of the ECS may represent a promising approach for the treatment of OMP frequently observed in patients with TMD.

## Introduction

Orofacial pain is a global term that comprises a category of disorders that include neuropathic pain, neurovascular disorders, and musculoskeletal disorders such as temporomandibular disorders (TMD) (1). Musculoskeletal pain affecting the masticatory muscles, known as orofacial musculoskeletal pain (OMP), is considered a painful TMD subtype and represents one of the leading causes of pain in the orofacial region. It is characterized by hypersensitivity in the masseter and/or temporalis muscles and this condition can impair essential functions of the stomatognathic system, such as chewing and speech. The development and severity of this condition can be influenced by different situations, like anatomical or biomechanical factors, sleep disturbances, sedentarism, and stress (2).

According to Svensson (1), painful TMD is typically chronic, bilateral, diffuse, and associated with jaw movement, aligning with the criterion for nociplastic pain addressed by Kosek et al. (2). Nociplastic pain is characterized by altered nociception without clear evidence of tissue damage or dysfunction of the somatosensory system and is often associated with central sensitization, a process involving amplification of the central nervous system's response to painful stimuli. Generalized pain sensitization, as seen in conditions such as fibromyalgia, chronic headaches, and TMDs, highlights the role of dysfunction in central inhibitory and facilitatory pathways in pain processing. Patients with nociplastic pain often experience multiple widespread pain, fatigue, sleep disturbances, cognitive difficulties, and various somatic symptoms, in addition to high levels of emotional distress, anxiety, depression, and pain catastrophizing (1,2).

Clinical trials on orofacial nociplastic pain conditions, such as painful TMD, identify stress as a predictor factor for pain, suggesting that psychosocial factors interact with other variables, worsening pain in the facial region (3). Emotional stress (ES) is a prevalent medical condition that provokes adaptive reactions controlled by neurohormonal processes aimed at maintaining physiological integrity. Increasing evidence indicates that ES can influence both the onset and maintenance of orofacial pain. This interplay between psychosocial stressors and pain highlights the involvement of central neural mechanisms, supporting hypotheses centered on sensitization processes (2,4,5).

The association between stress and orofacial pain suggests that central sensitization may be a key underlying mechanism of OMP, thus justifying the search for targeted modulatory treatments. This range of manifestations reinforces the hypothesis of altered central pain mechanisms, such as reduced descending inhibitory modulation and increased pain facilitation. In this context, endocannabinoid system modulators, such as phytocannabinoids or synthetic cannabinoids, have gained prominence as a potential strategy for the treatment of chronic nociplastic pain (5-7).

The endocannabinoid system is involved in the pain descending modulatory system with neurochemical pathways that inhibit pain sensation and is activated during stress (8). This system consists of cannabinoid receptors (CB_1_ and CB_2_), endogenous ligands (anandamide, 2-AG, and others), and enzymes involved in their biosynthesis and degradation. Endocannabinoids are retrograde signaling messengers at glutamatergic and GABAergic synapses and modulate the postsynaptic transmission, which interact with other neurotransmitters. They are involved in several physiological and pathological functions, such as: immunomodulation, inflammation, and analgesia (9,10).

In this context, the antinociceptive effects of the endocannabinoid system in orofacial pain have been increasingly recognized in recent years (9,11). Traditional treatments for chronic pain, particularly nociplastic pain, show limited efficacy, which highlights the need to identify alternative therapeutic options. Moreover, the endocannabinoid system is also involved in neural modulation during the systemic stress response (8,12). Preclinical studies suggest that the endocannabinoid system may play a crucial role in maintaining homeostasis by counteracting exaggerated behavioral responses to stress (13).

Although it is challenging to mimic TMD in preclinical research due to its nociplastic nature, experimental models involving emotional stress with orofacial musculoskeletal repercussions may offer a viable alternative. Therefore, the aim of this study was to better understand OMP induced by psychological/emotional stress, focusing on the role of the endocannabinoid system through the modulation of CB_1_ and CB_2_ cannabinoid receptors.

## Material and Methods

### Animals and ethical aspects

Wistar male rats (8 weeks old; 250 to 300 g), provided by the Central Animal Facility of Federal University of Ceará, were used in this study. The rats were randomly allocated into experimental groups (6-8 rats/group) in appropriate cages (3 animals per cage) in a special room under controlled temperature (24±2°C) and a 12-h light/dark cycle, with solid food and water *ad libitum*. This study was conducted following the ARRIVE guidelines. The experimental protocol was analyzed and approved by the Ethics Committee on Animal Research of the Federal University of Ceará (protocol 35/2013).

In this study, the sample size was calculated to detect a 20% difference in the intragroup head-withdrawal response (primary outcome) with a statistical power of 80%. The standard deviation of the mean and confidence interval were set at 15 and 95%, respectively. Based on these parameters, the estimated sample size was 8 animals per experimental group. Based on the methodological requirements for the induction of OMP through emotional stress using the communication box, a well-established model in Wistar rats (14), 8 animals are placed inside the individual compartments of the communication box during the protocol to ensure effective stress induction. The communication box contains 16 compartments: 8 housing electric foot-shock (EFS) animals that served exclusively as stress inducers (not included in the experimental groups and not subjected to any experimental evaluations) and 8 housing experimental animals. Failure to meet this distribution compromises the effectiveness of the stress protocol. Humane endpoints criteria were predefined, and although no animals required euthanasia for this reason, all animals were continuously monitored throughout the protocol for signs of extreme distress or suffering. After the triage phase (animals that responded to the von Frey stimulation with a clear head withdrawal response), animals were randomly assigned to either the Control (not exposed to emotional stress) or emotional stress (ES) condition using a computer-generated randomization list. Blinding was ensured throughout the experiment, with researchers responsible for drug administration and ES induction being distinct from those conducting the behavioral assessments.

### Cannabinoid drugs

CB_1_ (AM251; Tocris Biosciense, UK) or CB_2_ (AM630; Cayman Chemical, USA) receptor-selective antagonists (11) was dissolved in 4% dimethylsulfoxide (DMSO) and diluted in sterile 0.9% saline solution. A non-selective cannabinoid receptor agonist (WIN55,212; Cayman Chemical) was dissolved in absolute ethanol (5 mg:1 mL) (15). AM251 or AM630 (1 mg/kg) was administered on D4, and WIN55,212-2 (1 mg/kg) was administered on D11 of the emotional stress induction, 30 min before the stimulus in the communication box. The doses of the CB_1_ and CB_2_ antagonists (1 mg/kg) and the agonist (1 mg/kg) were selected based on previous studies demonstrating their efficacy in modulating nociceptive responses in rodents (11,15).

### Emotional stress experimental model

The communication box was used as an instrument to induce ES (14). The box consists of 16 compartments (16×16 cm) separated by transparent acrylic plates with several small holes. The plates prevent physical contact among the animals while allowing visual, auditory, and olfactory sensations of neighboring animals. The compartments were equipped with a grid floor of stainless-steel rods. An electro-stimulator (LE 12406, Panlab Harvard Apparatus, USA) of direct current (1 Hz) was connected to the grid to generate an electric current of 40 V to generate an electric foot-shock for 10 s with an interval of 60 s. The floors of eight compartments were covered by plastic plates to prevent electric stimulation and served as a non-shock compartment for the animals of the ES groups. Animals were divided into Electric Foot-Shock (EFS) groups and experimental groups. The ES group in the non-shock compartment were exposed to emotional stimuli from the neighboring animals (EFS group), such as vocalizations, urine or feces smell, and jump response. The control group was also confined in the non-shock compartment, but the electro-stimulator remained turned off.

The animals were first confined in compartments of the communication box for one hour without any electric foot-shock for 5 days in order to adapt to the environment. After the adaptation period, ES was induced for 14 days (D1-D14). Body weight gain was recorded on the first day (D1), before the stimulus, on the seventh day (D7), and on the fourteenth day (D14) of the ES. During the experimental protocol, the animals were evaluated daily for spontaneous behavior, breathing, posture, and facial expression, which were used as humane endpoint criteria.

### Behavioral tests

#### Masticatory muscles mechanical sensitivity test

The sensitivity of masticatory muscles was assessed by recording the force applied to the masseter and temporalis region required to elicit a reflex response (head withdrawal). Measurements were taken using a digital instrument by an examiner who was not informed of the treatment groups. For this, the electronic von Frey apparatus (Digital Analgesiometer, Insight, Brazil) was used. The apparatus is a force transducer that measures the pressure in grams (g), applied perpendicularly to the evaluated region. The reflex response to pressure is expressed as head withdrawal response (g), which reflects the nociceptive threshold. The animals were exposed to a five-day habituation period in the test room for head-withdrawal threshold measurements, under standardized temperature and reduced illumination.

The animals were kept for 10 min in plastic boxes and submitted to the application of the Von Frey apparatus in the region of the left masticatory muscles. An observer was trained to apply a gradual pressure to the region and the apparatus was automatically removed when the animal withdraw the head (16,17). Triplicate measurements of force thresholds were taken from the masseter and temporalis muscles. The baseline test was performed on D1 of the ES protocol, before the exposure to the communication box. The test was performed on D4, D8, D11, and D14, 90 min after the communication box protocol. The animals' spontaneous behavior was respected throughout the procedure, with sufficient intervals between measurements to allow free movement within the cage. The animals were gently restrained only during the assessment of the nociceptive threshold.

The results were registered as the delta of force (g) calculated by subtracting the value (g) obtained on D4, D8, D11, and D14 from the value on D1 (before the emotional stress protocol).

#### Elevated plus-maze test

To assess anxiety-like behavior, the elevated plus-maze test (16) was performed on D14, 30 min after the communication box protocol. For this, the animals were randomly placed at the center of the plus maze and observed for 5 min to record the number of entries into the open arms and the time spent in the open arms. The time spent in the open arms was converted to percentage (time in seconds in the open arms × 100/total observation time).

### Adrenal histological analysis

Histological analysis was performed in adrenal gland samples, harvested on D14. For this, sixteen rats were anesthetized with an intraperitoneal injection of ketamine (100 mg/kg) and xylazine (10 mg/kg) and intracardially perfused. Adrenal glands were fixed in 10% formaldehyde for 24 h, then placed in 70% alcohol for another 24 h, and processed for paraffin inclusion. Serial sections (4-μm thickness) were obtained and stained with hematoxylin and eosin. Slides were examined and photographed under a microscope (Leica DM 2000, Leica, Germany) with 400× and 1000× magnification. In the adrenal glands, the zona fasciculata region was observed in the cortex, quantifying the area of cytoplasmic vacuoles with of 1000× magnification using the ImageJ software (NIH, USA). The color threshold defined the higher and lower limits to selected and unselected pixels. Results are reported in percentage of the total positive cytoplasmic vacuoles area (selected pixels).

### c-Fos, CB_1_, and CB_2_ immunofluorescence assay

Immunofluorescence analyses were performed in the masseter muscle, TG, and Sp5C sections, which were harvested on D14. The TG and Sp5C areas were dissected, placed in 4% paraformaldehyde (PFA) (Sigma-Aldrich^®^, USA) for 2 h and cryoprotected in a 30% sucrose solution (Dinâmica Química Contemporânea Ltda., Brazil) for 72 h. The collected tissues were embedded in Tissue-tek O.C.T. compound (Sakura^®^, Netherlands) and stored at −80°C. Serial sections (10-μm thickness) for the immunofluorescence assay were performed in a cryostat (Leica CM1850, Leica) at -24°C. The sections used in the study were cut at -13.92 mm to -15.48 mm from bregma. Masseter muscle samples were fixed in 10% formaldehyde for 24 h, then placed in 70% alcohol for another 24 h and processed for paraffin inclusion. Serial sections (4-μm thickness) were obtained for the immunofluorescence protocol. Sections were fixed in methanol (Vetec Química Fina Ltda., Brazil), and the antigenic recovery was performed (95°C) in 0.1 M (pH 6.0) citrate buffer. Subsequently, the nuclear membrane was permeabilized with 0.2% triton X-100 (in c-FOS slides) and blocked with 5% bovine serum albumin and 0.3 M glycine in all slides. Sections were incubated (4°C) overnight with the primary antibody rabbit anti-c-Fos (Santa Cruz Biotechnology^®^; Cat# sc-253; RRID: AB_2231996, USA), rabbit anti-CB_1_ (Abcam^®^, UK; #Cat ab23703; RRID: AB_447623); or rabbit anti-CB_2_ (Santa Cruz Biotechnology^®^; #Cat sc-25494; RRID: AB_2082784) in a 1:200 dilution. After this, sections were incubated (room temperature) with secondary antibody donkey anti-rabbit IgG Alexa fluor 568 (Invitrogen^®^; Cat# A10042, RRID: AB_2534017; 1:400, USA). For the labeling of neuron cell bodies in nervous tissue samples, sections were incubated with mouse anti-NeuN antibody conjugated with Alexa fluor 488 (Merck Millipore^®^; Cat# MAB377X, RRID: AB_2149209; 1:100, USA). Tissue sections were incubated for 30 min with DAPI (4,6′-diamidino-2-phenylindole) (Invitrogen^®^; D1306, RRID: AB_2629482) (4 µL in 200 mL of PBS) to label the cell nucleus. Then, the slides were mounted with ProLong Gold Antifade Mountant (Invitrogen^®^) and photographed in a laser scanning confocal microscope (Zeiss LSM 710, Carl Zeiss, Germany). The fluorescent area in the photos was quantified by differentiating the fluorescent areas (pixels) by the greater color saturation, associated with the fluorescence (red or green). For this, the program Fiji ImageJ (NIH) was used by a blinded examiner. The color threshold defined the upper and lower limits for the positive fluorescent area. Two sections from the TG and Sp5C regions of at least 6 animals per group were evaluated and the results are reported in percentage of the total positive fluorescent area (Alexa Fluor 568) in relation to NeuN (Alexa Fluor 488) positive fluorescent area. Two sections from the masseter muscle of at least 6 animals per group were evaluated and the results are presented in percentage of the total positive fluorescent area (Alexa Fluor 568) in relation to total image area.

### Experimental protocol

The experimental protocol was divided into Protocol I and Protocol II.

Protocol I was designed to evaluate nociceptive behavior induced by emotional stress through the masticatory muscles mechanical sensitivity test, anxiety-like behavior through the elevated plus-maze test, c-Fos expression in areas of the trigeminal nociceptive pathway (TG and Sp5C), and cannabinoid receptors in these same areas, as well as in the masseter muscle. Systemic response to stress was evaluated by adrenal histological analysis and weight gain. For this, sixteen rats were randomly divided into 2 groups (n=8/group): Control group and ES.

Protocol II aimed to investigate the effect of the endocannabinoid system pharmacological modulation. For this, 96 rats were randomly divided into 12 experimental groups (n=8/group), allocated into two main conditions: Control and ES. Each condition was subdivided into six groups: three groups received pharmacological treatment with AM251, AM630, or WIN55,212-2, and three groups received only the respective vehicle solution. Therefore, the Control groups consisted of: Control + AM251, Control + AM630, Control + WIN55,212-2, Control + Vehicle of AM251, Control + Vehicle of AM630, and Control + Vehicle of WIN55,212-2. Similarly, the Emotional Stress (ES) groups consisted of: ES + AM251, ES + AM630, ES + WIN55,212-2, ES + Vehicle of AM251, ES + Vehicle of AM630, and ES + Vehicle of WIN55,212-2. In these groups, nociception was evaluated by the mechanical sensitivity test of masticatory muscles. Supplementary Figure S1 illustrates the steps described in both protocols.

### Statistical analysis

Initially, data normality was checked using the Shapiro-Wilk test. Data are reported as means±SE. Statistical analyses of the data from the masticatory muscle mechanical sensitivity test were performed by two-way ANOVA followed by Tukey's test. For other data, Student's *t*-test was used and statistical significance was considered when P<0.05. All analyzes were performed using the GraphPad Prism version 9.0 for Mac (GraphPad Software, USA).

## Results

### Effects of emotional stress on adrenal glands and body weight gain

**Figure 1:**
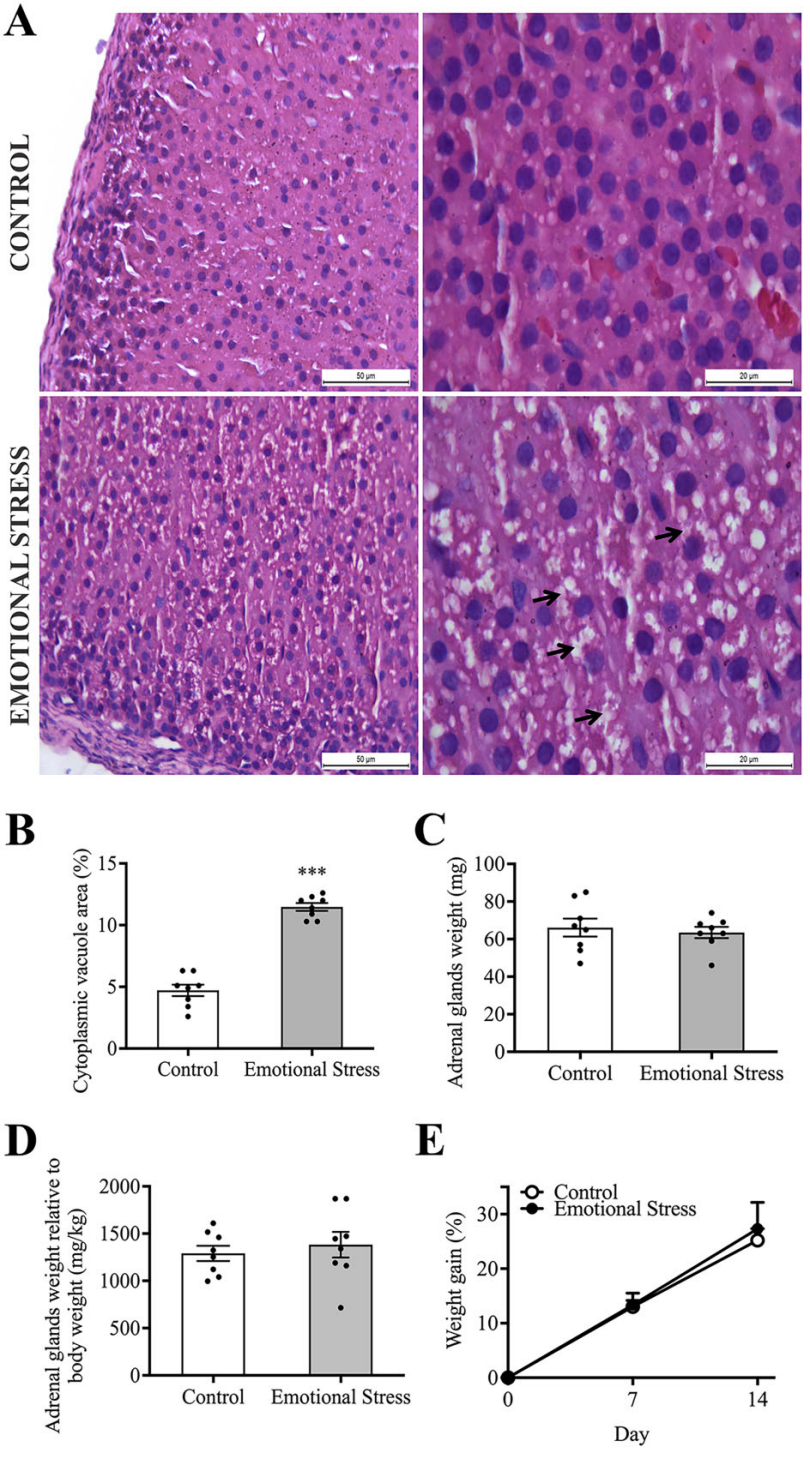
Effect of emotional stress on adrenal glands and body weight gain. **A**, Photomicrographs of hematoxylin and eosin staining showing the *zona fasciculata* of the adrenal gland cortex (magnification of 400× and 1000×, scale bars 50 and 20 μm) of rats subjected to emotional stress and animals not submitted to emotional stress (control), showing cytoplasmic vacuoles (arrow) mainly in the emotional stress group. **B**, Cytoplasmic vacuole area in the *zona fasciculata* of the adrenal gland cortex. **C**, Gross weight of the adrenal glands. **D**, Adrenal gland weight relative to body weight. **E**, Body weight gain. Data are reported as means±SE. ***P<0.001; unpaired *t*-test and (**D**) two-way ANOVA, Tukey post-test.

The analysis of cytoplasmic vacuoles in the adrenal gland cortex fasciculata zone showed distinct profiles in the ratio of cytoplasmic vacuole area to total area (%). The ES group showed a 90.94% higher concentration of cytoplasmic vacuoles (10.03±0.52), while the control group (5.25±0.38) showed a basal content of these vacuoles (P<0.0001; Figure 1A and B). ES had no effect on adrenal gland weight (P=0.624) (Figure 1C and D). There were no statistical differences in the animals' body weight among the ES and control groups (P=0.734) (Figure 1E).

### Emotional stress induced anxiety-like behavior and masticatory muscle sensitivity

**Figure 2:**
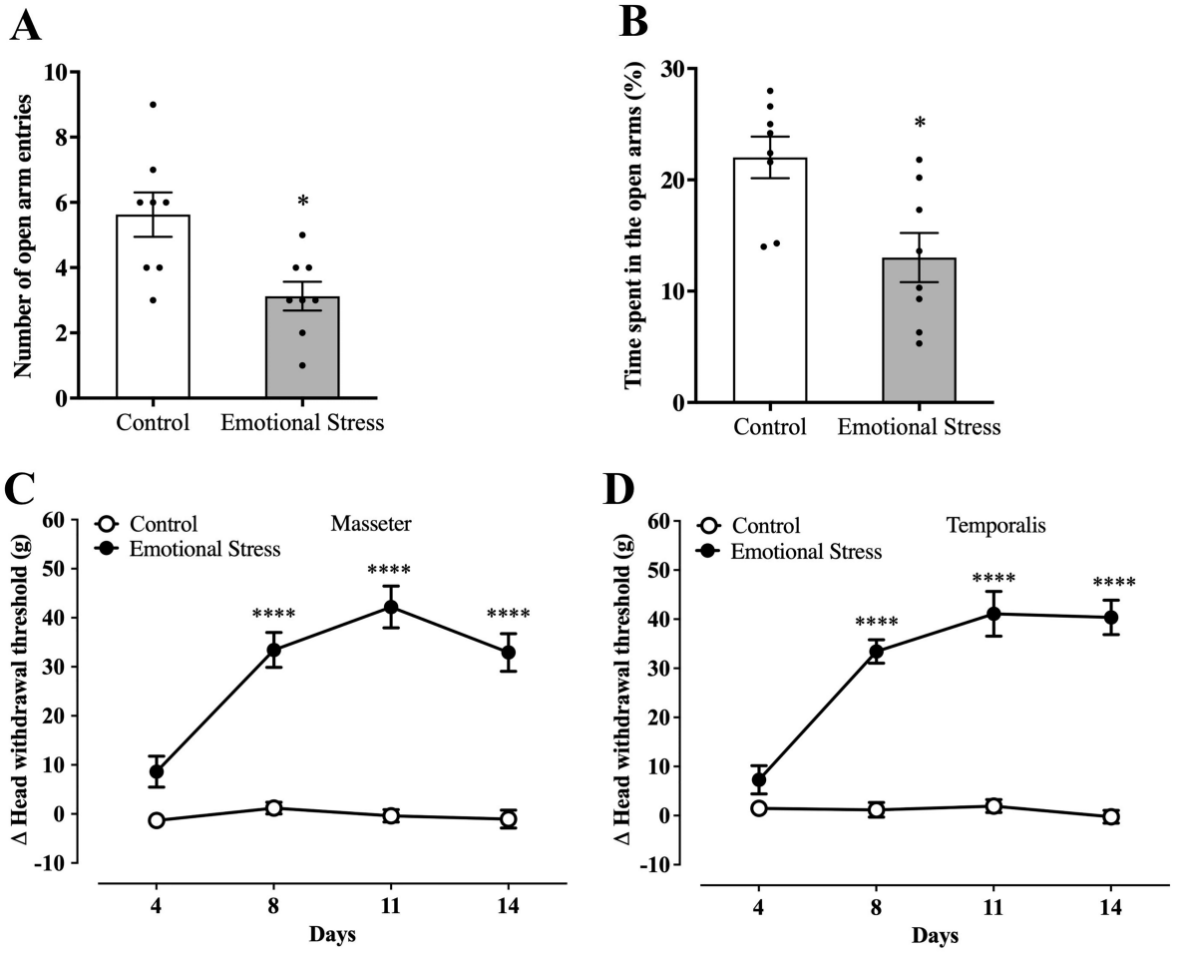
Effect of emotional stress on the plus maze test and on mechanical sensitivity of masseter and temporalis muscles in rats. **A**, Number of open arm entries in plus maze test. **B**, Time spent in the open arms (%) of plus maze test. The time course of the increase in mechanical sensitivity (delta head withdrawal threshold in the von Frey test) in masseter (**C**) and temporalis (**D**) muscles. Data are reported as means±SE; *P<0.05; unpaired *t*-test (**A** and **B**) and ****P<0.0001 *vs* control; two-way ANOVA, Tukey post-test (**C** and **D**).

The plus maze test showed that the animals of the ES groups had fewer entries into the open arms (P=0.022) (Figure 2A) and less time spent in the open arms (P=0.025) (Figure 2B).

The results involving mechanical sensitivity of the masticatory muscles are expressed as a delta (Δ) of the head withdrawal response threshold in grams, calculated by the difference between the nociceptive threshold measured on D1 (before stress induction) and the other experimental days. Thus, the intensity of sensitivity was proportional to the delta value. Figure 2 shows that ES increased the Δ head withdrawal threshold of the masseter (Figure 2C) and temporalis (Figure 2D) muscles from D8 compared with the control group (P<0.0001). The nociceptive response remained until D14 of the emotional stress induction (P<0.0001).

### Emotional stress increased c-Fos density in TG and spinal trigeminal nucleus caudalis (Sp5C)

**Figure 3:**
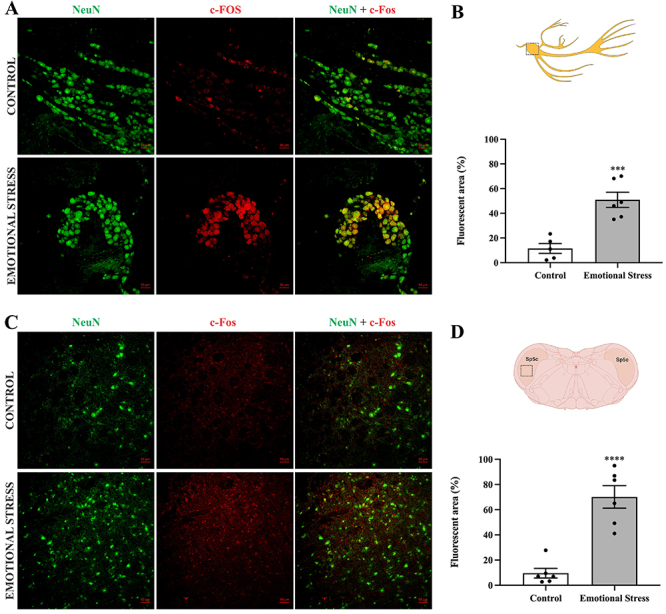
Emotional stress increases c-Fos expression in trigeminal ganglia (TG) and in spinal trigeminal nucleus caudalis (Sp5C). Confocal photomicrography of the TG (**A**) and Sp5C (**C**): NeuN (neuronal marker) shown in green, c-Fos shown in red, merge shown in yellow. Magnification: 200×; scale bar: 50 µm. **B**, Quantification of the fluorescent areas of the c-Fos expression in TG. **D**, Quantification of the fluorescent areas of the c-Fos expression in Sp5C. Data are reported as means±SE. ***P<0.001, ****P<0.0001; unpaired *t*-test.

Results showed that ES increased the neuronal c-Fos positive area (%) by immunofluorescence staining in both TG (P=0.0006) (Figure 3A and B) and Sp5C (P<0.0001) (Figure 3C and D) areas compared to the control group.

### CB_1_ and CB_2_ agonists and antagonists affected the emotional stress-induced masticatory muscle mechanical sensitivity

**Figure 4:**
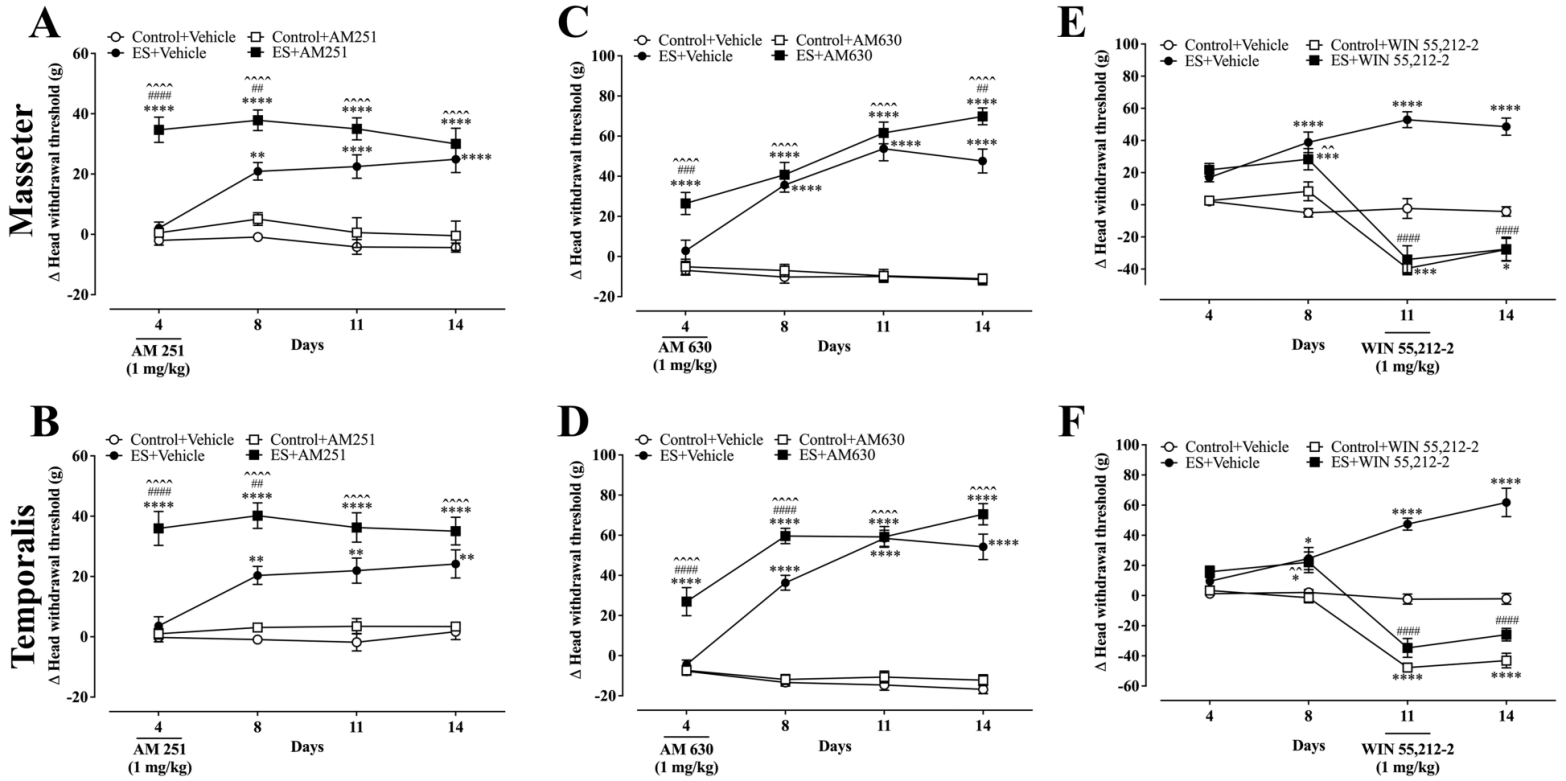
Effect of AM251, AM630, and WIN55,212-2 on emotional stress (ES)-induced mechanical sensitivity of masseter (**A**, **C**, **E**) and temporalis muscles (**B**, **D**, **F**) of rats. Data are reported as means±SE. *P<0.05, **P<0.01, ***P<0.001, ****P<0.0001 *vs* control + vehicle; ^##^P<0.01, ^###^P<0.001, ^####^P<0.0001 *vs* ES + vehicle; ^ˆˆ^P<0.01, ^ˆˆˆˆ^P<0.0001 *vs* control + drug [AM251 or AM630 or WIN55,212-2]; two-way ANOVA, Tukey post-test. AM251: selective CB_1_ receptor antagonist; AM630: selective CB_2_ cannabinoid antagonist; WIN55,212-2: non-selective cannabinoid receptor agonist.

The CB_1_ or CB_2_ receptor antagonists increased the ES-induced mechanical sensitivity in both the masseter and temporalis muscles on D4 (P<0.0001) and D5 (P<0.01; P<0.0001, respectively) experimental days (Figure 4A and B). AM251 did not alter the Δ head withdrawal response in rats not submitted to the ES protocol (Control+AM251). The selective CB_2_ antagonist AM630 increased the ES-induced mechanical sensitivity in both the masseter and temporalis muscles (Figure 4C and D). The differences between the ES and ES+AM630 groups were observed on D4 and D8 for the temporalis muscle and only on D4 for the masseter muscle (P<0.0001). AM630 also did not alter the Δ head withdrawal response in rats not submitted to ES (Control+AM630).

The cannabinoid receptor non-selective agonist WIN55,212-2 (1 mg/kg; *ip*, single injection on D11) decreased the ongoing ES-induced mechanical sensitivity in both the masseter and temporalis muscles (P<0.0001) (Figure 4E and F). The differences between ES and ES+WIN55,212-2 groups were observed on D11 (injection day; P<0.0001) and D14 (P<0.0001). WIN55,212-2 also altered the Δ head withdrawal response in rats not submitted to ES (Control+WIN55,212-2) when compared to the control group (Control+Vehicle) on D11 (injection day; P<0.0001) and D14 (P<0.01) (Figure 4E and F).

### Effect of emotional stress on CB_1_ and CB_2_ density

**Figure 5:**
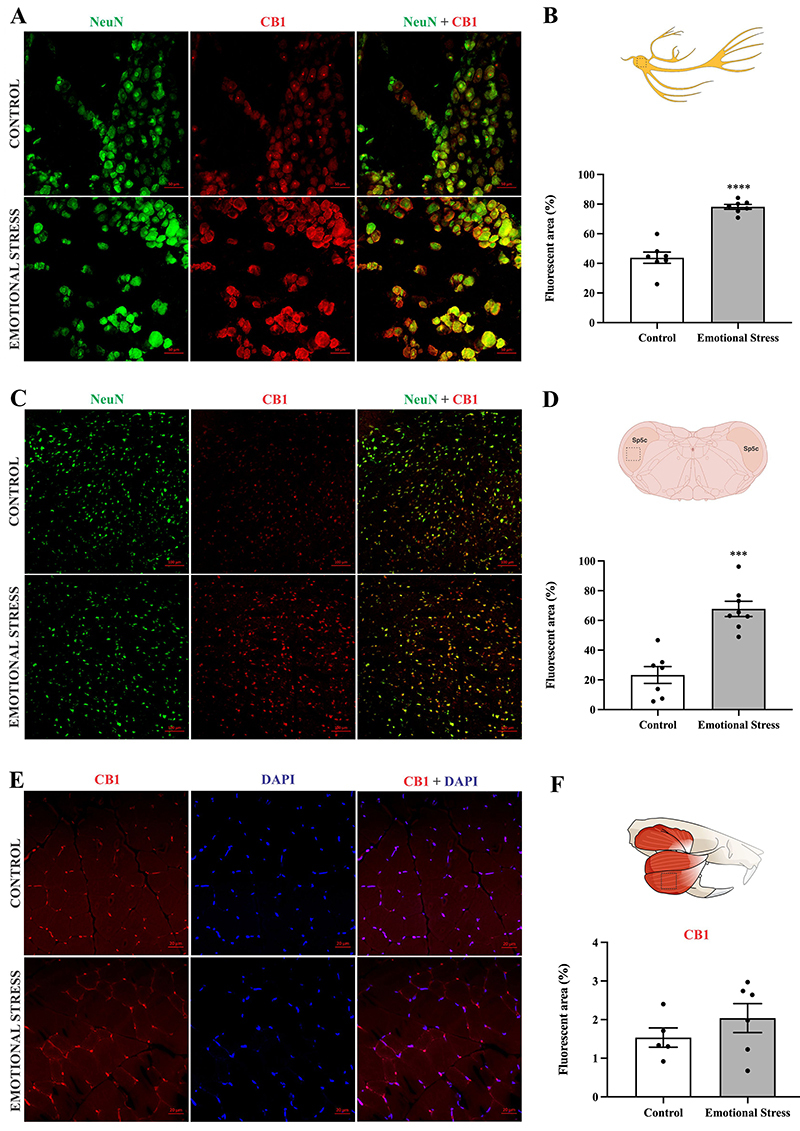
Effect of emotional stress on CB_1_ expression in trigeminal ganglia (TG), spinal trigeminal nucleus caudalis (Sp5C), and masseter muscle. Confocal photomicrography of TG (**A**), Sp5C (**C**), and masseter (**E**). Panels **A** and **C**: NeuN (neuronal marker), green; CB_1_, red; merge, yellow. Panel **E**: CB_1_, red; DAPI, blue; merge, magenta. Magnification: 200×; scale bar: 50 µm. Quantification of the fluorescent areas of CB_1_ expression in TG (**B**), in Sp5C (**D**), and in masseter muscle (**F**). Data are reported as means±SE. ***P<0.001, ****P<0.0001; unpaired *t*-test.

**Figure 6:**
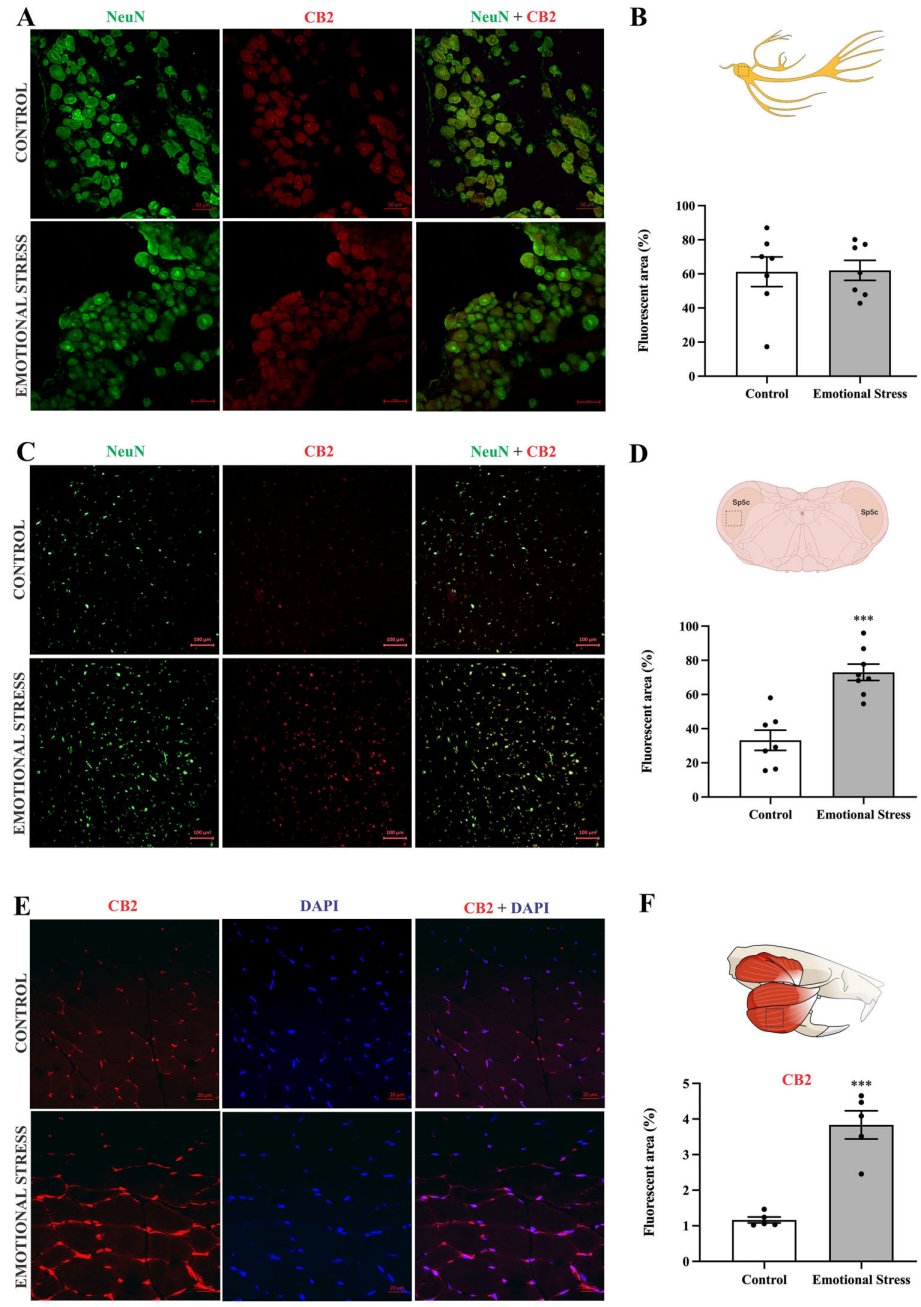
Effect of emotional stress on CB_2_ expression in trigeminal ganglia (TG) and in spinal trigeminal nucleus caudalis (Sp5C), and masseter. Confocal photomicrography of the TG (**A**), Sp5C (**C**), and masseter (**E**). Panels **A** and **C**: NeuN (neuronal marker), green; CB_2_, red; merge yellow. Panel **E**: CB_2_, red; DAPI, blue; merge, magenta. Magnification: 200×; scale bar: 50 µm. Quantification of the fluorescent areas of the CB_2_ expression in TG (**B**), in Sp5C (**D**), and in masseter muscle (**F**). Data are reported as means±SE. ***P<0.0001, unpaired *t*-test.

ES induced an increase in CB_1_-positive area (%) by immunofluorescence staining in TG (P<0.0001) (Figure 5A and B) and in Sp5C sections (P=0.0006) (Figure 5C and D). When evaluating the positive area for CB_2_, the results were similar in Sp5C (P<0.0001) (Figure 6C and D), but the expression of CB_2_ in TG was not different between the groups (P=0.936) (Figure 6A and B). In the masseter muscle, ES increased CB_2_-positive area (P=0.0002) (Figure 6E and F), but not CB_1_-positive area (P=0.313) (Figure 5E and F) compared to the control group.

## Discussion

This study demonstrated that ES induced mechanical sensitization in the masseter and temporalis muscles. This sensitization was evidenced by reduced response thresholds to the electronic von Frey test and a concomitant increase in c-Fos expression within the TG and the Sp5C, indicating activation of the trigeminal pain pathway. Furthermore, animals subjected to ES exhibited upregulated expression of both cannabinoid receptors CB_1_ and CB_2_ throughout this neural pathway. The functional relevance of this upregulation was confirmed pharmacologically, as the administration of CB_1_/CB_2_ receptor agonists and antagonists significantly modulated the stress-induced nociceptive behavioral responses.

The communication box is an experimental model used to investigate stress induced by auditory, olfactory, and visual exposure to other animals undergoing distress (14,16). This model has been shown to be effective to elicit systemic stress responses, such as increased plasma corticosterone and adrenocorticotropic hormone (ACTH) levels (18,19) and behavior changes in the elevated plus maze apparatus (16). In our study, the ES group animals presented stress signals confirmed by the plus-maze test, as evidenced by a reduced number of entries into the open arms.

Another parameter used to assess stress was the evaluation of the adrenal glands. Stressful situations activate the HPA axis, leading to stimulation of the zona fasciculata and production of glucocorticoid (20). Results showed an increased number of cytoplasmic vacuoles in the zona fasciculata of the adrenal gland cortex in the ES group. These vacuoles may be related to changes in lipid enzymatic activity in the adrenal glands associated with an increased synthesis of corticosterone, a hormone related to stress in rodents (20,21). Interestingly, body weight gain and adrenal gland gross weight were not significantly different between Control and ES groups, which can be related to a short ES induction time.

The model by Rosales et al. (14), which was used in this study, evaluates the impact of ES using the communication box to promote hyperactivation in the masticatory muscles, suggesting a bruxism-like effect. In addition, another study (16) demonstrated changes in the masticatory muscle nociceptive threshold. Accordingly, this work focused on the orofacial region, and nociceptive thresholds in other regions of the body were not assessed. This represents a limitation of this study, as it cannot be determined whether this model of ES selectively affects the masticatory muscles or also influences nociception in other regions.

Stressed animals developed changes in the mechanical threshold of masticatory muscles, showing nociceptive behavior after 8 experimental days and lasting until the last day of the experimental protocol, with a peak in D11. The study of Huang et al. (16) concluded that stressed animals showed a decrease in the masticatory muscle nociceptive threshold, but with a peak on D7, a difference that may be associated with the animal strain used. The authors used Sprague-Dawley rats, which are more sensitive to mechanical stimuli than Wistar rats and other species. The authors also showed that mechanical sensitivity in the masticatory muscles is associated with stress exposure, since the mechanical threshold returns to baseline 14 days after the end of the experimental stress protocol.

The results shown here suggest that emotional stress activates the trigeminal nociceptive pathway, which is corroborated by the increase in c-Fos immunostaining observed in the TG and Sp5C nucleus. The TG contains the cell bodies of primary sensory afferent neurons, while the Sp5C area within the medulla oblongata constitutes the principal site of synaptic relay between afferent neurons and second-order central neurons of the trigeminal nociceptive pathway. This circuitry mediates orofacial pain transmission arising from masticatory muscles, particularly the temporalis and masseter. Other preclinical studies have shown that psychological stress induces masticatory muscle mechanical sensitivity in animal models (16,22); however, these studies did not assess c-Fos labeling in the trigeminal nociceptive pathway. The increased expression of c-Fos, a well-established marker of neuronal activation (23), in these regions corroborates the neuronal activation in the trigeminal pathway (24).

In the present study, the pain in the masticatory muscles that developed from the 8th experimental day onward was exclusively induced by emotional stress. Notably, no hyperalgesic or phlogistic substances were injected into the masticatory muscles. Thus, this model intends to simulate alterations in the brain regions involved in pain processing, stimulating a central sensitization process that is directly related to the pathophysiology of nociplastic pain. Chronic stress is closely related to this type of pain condition (25).

In the same manner, the origin of symptoms in patients with painful TMD may not always be attributed to local anatomical alterations. Psychosocial factors must also be considered, as they are directly related to the neurobiology of pain. Supporting this view, painful TMD are classified as a form of nociplastic pain, as described by Svensson (1) and consistent with the altered nociception criteria proposed by Kosek et al. (2), where alterations in pain processing, such as central sensitization, play a pivotal role in the underlying mechanism.

Extending this perspective to the epidemiological context, TMD has recently been reported to have an incidence of 34% in the world population, and the majority of affected people have pain related to masticatory muscles (OMP), which is considered the main type of non-odontogenic orofacial pain (4,26). In this clinical condition, stress can be a predicting factor. Furthermore, individuals with OMP have high levels of anxiety, depression, and catastrophization, which are factors closely related to stress. Psychological or emotional stress is clearly implicated in the etiology of OMP (27), initially functioning as a systemic adaptive response, but leading to the maintenance of pain in the long term. Additionally, stress increases sympathetic activity and contributes to changes in pain threshold, influencing the descending pain modulatory systems (28).

TMD in women is possibly influenced by sex hormones and biopsychosocial factors (4,26). Sex-related neurobiological differences are known to influence stress-induced analgesia and pain responses, but these differences were not studied in the present work which, to the best of the authors' knowledge, is the first to investigate the relationship between emotional stress, masticatory muscle pain, and the endocannabinoid system in rats. To ensure greater control of experimental variables and improved standardization of the evaluated groups, the present study was conducted using only male animals. This methodological choice represents a limitation of the study and highlights the importance of including females in future investigations.

The descending pain modulatory system operates through several mechanisms, including the opioid and endocannabinoid systems. The ECS comprises endogenous ligands (anandamide and 2-AG), cannabinoid receptors, and enzymes responsible for their synthesis and degradation (9). In this sense, cannabinoid receptors CB_1_ and CB_2_ are pivotal in the regulation of physiological pain, exerting their effects through the modulation of neuronal glutamate release and interactions with microglial cells, thereby maintaining a fine balance between excitatory and inhibitory neurotransmission (9).

Emerging evidence has proposed that nociplastic pain may be underpinned by an ECS deficiency, a framework that could account for the increased expression of cannabinoid receptors observed in our study within the TG and the Sp5C, both critical nodes for the activation and transmission of nociceptive signal in the masseter and temporalis muscles (29). Such upregulation may constitute a homeostatic response to persistent emotional stress, a well-recognized factor contributing to pain chronification. Additionally, it is known that nociplastic pain, in which central sensitization represents a key feature, involves microglial activation within the context of neuroinflammation, as well as glutamatergic changes among the mechanisms studied. The ECS may influence the development of these mechanisms, highlighting the need for future investigations that differentiate central and peripheral mechanisms (9).

Another example of chronic pain mediated by the trigeminal pathway is chronic migraine, a neurovascular disorder arising from abnormal sensory processing and involving both central and peripheral sensitization mechanisms. Recent studies have suggested that the ECS may represent a promising therapeutic avenue to mitigate the physiological and inflammatory components underlying migraine-related pain (30). Nevertheless, in painful TMD, these therapeutic benefits remain largely unsubstantiated, underscoring the relevance of our findings as an initial step toward elucidating the compensatory upregulation of CB_1_ and CB_2_ receptors and their modulatory role in orofacial pain. Moreover, the involvement of the ECS in stress processing may further reinforce its contribution to the maintenance of maladaptive mechanisms associated with the transition from acute to chronic pain states (29).

Our study focused on CB_1_ and CB_2_ receptor modulation, with the absence of direct biochemical assessment of anandamide and 2-AG representing a limitation. Future studies may incorporate endocannabinoid quantification to further elucidate system dynamics in stress-induced orofacial pain, which would help to determine whether the observed upregulation of CB_1_ and CB_2_ receptors reflects a compensatory response or an endocannabinoid system deficiency.

The data showing the upregulation of CB_1_ and CB_2_ receptors in trigeminal pain-processing areas, prompted us to investigate whether these receptors participated in the modulation of OMP induced by ES. Thus, cannabinoid receptor antagonists (AM251 and AM630) and non-selective cannabinoid receptor agonist (WIN55,212-2) were administered to investigate if the blockade or activation of CB_1_ and CB_2_ receptors would modify the painful response. The findings showed that a single administration of WIN55,212-2 inhibited ongoing mechanical sensitivity on masticatory muscles caused by ES, and the analgesic effect remained until the last experimental day. Interestingly, the cannabinoid agonist increased the nociceptive threshold suggesting a hypoalgesic effect in the non-stressed group. Although WIN55,212-2 caused this effect, it does not appear to be a cannabimimetic effect, as other studies showed that the utilized dose (1 mg/kg) did not promote cannabimimetic effects (31,32).

Conversely, the blockade of the cannabinoid receptors, CB_1_ and CB_2_, by the acute administration of the respective selective antagonists, AM251 and AM630, provoked an increase in the mechanical sensitivity associated with ES, anticipating the nociceptive state to the 4th experimental day of the stress protocol and increased mechanical sensitivity on the 8th day. This suggests that cannabinoid receptor blockade possibly prevented endocannabinoids modulation of the nociceptive threshold.

An additional finding was that the effect of AM251 was longer lasting when compared to AM630, especially in the masseter muscle, suggesting a greater participation of CB_1_ in this process. In line with this observation, previous studies indicate a greater participation of CB_1_ than CB_2_ receptors in pain pathway modulation (8,9). Corroborating these results, CB_1_ expression increases in both the TG and the Sp5C, whereas CB_2_ expression is upregulated only in the Sp5C nucleus. Other authors have also demonstrated that CB_1_ plays a critical role in mediating the effects of 2-AG on the glutamatergic system. In this context, elevated 2-AG levels are thought to exert a protective function by stimulating CB_1_ receptors, thereby restricting glutamate release and safeguarding the nervous system from the excitotoxic effects of this neurotransmitter (33). Corroborating these findings, Zoppi et al. (34) demonstrated the regulatory role of the CB_1_ receptor in stress-induced excitotoxicity and neuroinflammation. The results obtained by western blotting and PCR show an increase in CB_1_ protein expression in agreement with the increase in gene expression of its mRNA. The authors associate this increase of the homeostatic response to the excess glutamate that is normally released upon exposure to stress (35). Another study showed that rodents exposed to chronic unpredictable stress exhibited an increase in the maximum density of the CB_1_ receptor binding site in the prefrontal cortex, showing that this receptor is important in the homeostatic response to stress (35).

CB_2_ also appears to be differentially modulated depending on the type of pain. In an inflammatory pain model, its expression increases in peripheral sites such as DRG and peripheral nerve endings and, in a neuropathic pain model, it increases in DRG and spinal cord (36). An interesting finding was observed when evaluating cannabinoid receptor labeling in the masseter muscle of animals subjected to emotional stress. A higher density of CB_2_ labeling was found in stressed animals, while no changes were detected in CB_1_ labeling. Indeed, skeletal muscle expresses both types of receptors, as demonstrated in both humans and rodents (37). To our knowledge, no studies have assessed cannabinoid receptor expression in masticatory muscles. Thus, this would be the first study to evaluate differences in CB_1_ and CB_2_ receptor density in the masseter muscle of male rats with stress-induced OMP and to integrate emotional stress, OMP, and ECS. These findings highlight a potential nociplastic mechanism underlying stress-related OMP and suggest the ECS as a promising therapeutic target.

The cannabinoid receptor expression has been investigated in other muscles. Dalle and Koppo (38) reported higher CB_2_ expression in type II fibers and higher CB_1_ expression in type I fibers in the human vastus lateralis skeletal muscle of older men compared to younger. This is particularly interesting because type II fibers have low fatigue resistance and appear to be involved in the pathophysiology of bruxism. Studies suggest that bruxism may shift the composition of the masseter muscle toward a higher proportion of type II fibers (39). This shift could be related to increased mechanical demand on masticatory muscles during bruxism episodes, such as clenching or grinding (40). Thus, the increase in type II fibers might explain the higher CB_2_ density in the masseters of stressed animals, given that the communication box ES model not only enhances masseter mechanical sensitivity, but also increases masticatory muscle activity, mimicking bruxism (14). However, further experiments would be needed to confirm this correlation.

Here, we demonstrated that the non-selective agonist WIN55,212-2 effectively inhibited OMP, which may result from its action on CB_1_ and CB_2_ receptors in the trigeminal pain pathway. However, the fact that the masseter muscle expresses both CB_1_ and CB_2_ receptors, and that CB_2_ expression increases in stressed animals, suggests that WIN55,212-2 might also influence masticatory muscle contraction. This provides additional support for the potential use of cannabinoids in reducing pain and fatigue in patients with painful TMD. Furthermore, there is consistent evidence that the ECS is involved in the modulation of stress, plays a central role in pain control, and participates in the trigeminal pain pathway. These findings highlight a potential nociplastic mechanism underlying stress-related OMP and suggest the ECS as a promising therapeutic target.

**Figure 7:**
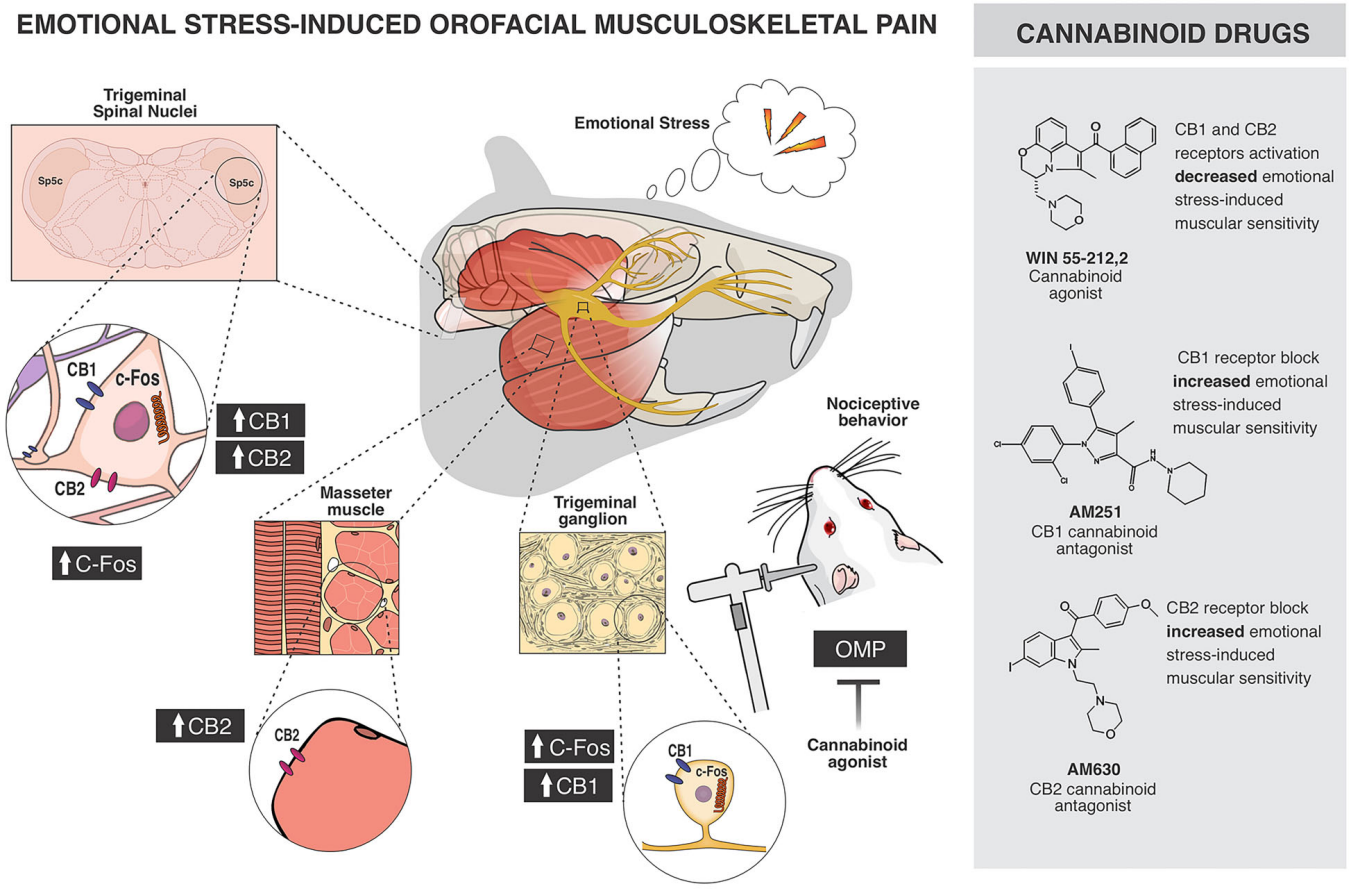
Emotional stress-induced orofacial musculoskeletal pain (OMP) is attenuated by the endocannabinoid system. Emotional stress induced pain in the masticatory muscles, as evidenced by increased mechanical sensitivity in the masseter and temporalis muscles, is accompanied by elevated c-Fos expression in the trigeminal ganglion (TG) and trigeminal nucleus caudalis (Sp5C). Pharmacological modulation with AM251 (a CB_1_ receptor antagonist) and AM630 (a CB_2_ receptor antagonist) increased OMP, whereas WIN55,212-2 (a non-selective cannabinoid receptor agonist) inhibited masticatory muscles mechanical sensitivity. Additionally, emotional stress increased CB_1_ receptor density in the TG, enhanced both CB_1_ and CB_2_ receptor densities in the Sp5C, and elevated CB_2_ receptor density in the masseter muscle.

In conclusion, the data suggest that ES causes mechanical sensitivity in the temporalis and masseter muscles, mimicking what happens in patients with painful TMD. ES also increases the expression of cannabinoid receptors in the trigeminal pain pathway and in the masseter muscle (Figure 7). The blockade or activation of these receptors, mainly CB_1_, modifies the sensitivity in the evaluated muscles, indicating that cannabinoid drugs or the modulation of the ECS may represent a promising therapeutic strategy for the treatment of stress-associated OMP, which is frequently observed in patients with TMD.

## Data Availability

All data generated or analyzed during this study are included in this published article.

## Supplementary Materials

Click to view [pdf].

## References

[1] Svensson P (2024). Could painful temporomandibular disorders be nociplastic in nature? A critical review and new proposal. Acta Odontol Scandi.

[2] Kosek E, Clauw D, Nijs J, Baron R, Gilron I, Harris RE (2021). Chronic nociplastic pain affecting the musculoskeletal system: clinical criteria and grading system. Pain.

[3] Fillingim RB, Ohrbach R, Greenspan JD, Knott C, Diatchenko L, Dubner R (2013). Psychological factors associated with development of TMD: the OPPERA prospective cohort study. J Pain.

[4] Fillingim R, Ohrbach R, Greenspan J, Sanders A, Rathnayaka N, Maixner W (2020). Associations of psychologic factors with multiple chronic overlapping pain conditions. J Oral Facial Pain Headache.

[5] Kosek E, Cohen M, Baron R, Gebhart GF, Mico JA, Rice ASC (2016). Do we need a third mechanistic descriptor for chronic pain states?. Pain.

[B06] Fitzcharles MA, Petzke F, Tölle TR, Häuser W (2021). Cannabis-based medicines and medical cannabis in the treatment of nociplastic pain. Drugs.

[7] Maglaviceanu A, Peer M, Rockel J, Bonin RP, Fitzcharles MA, Ladha KS (2024). The state of synthetic cannabinoid medications for the treatment of pain. CNS Drugs.

[8] Micale V, Drago F (2018). Endocannabinoid system, stress and HPA axis. Eur J Pharmacol.

[9] Zubrzycki M, Stasiolek M, Zubrzycka M (2019). Opioid and endocannabinoid system in orofacial pain. Physiol Res.

[10] Nadal X, La Porta C, Bura SA, Maldonado R (2013). Involvement of the opioid and cannabinoid systems in pain control: new insights from knockout studies. Eur J Pharmacol.

[11] Gondim DV, Costa JL, Rocha SS, Brito GAC, Ribeiro RA, Vale ML (2012). Antinociceptive and anti-inflammatory effects of electroacupuncture on experimental arthritis of the rat temporomandibular joint. Can J Physiol Pharmacol.

[12] Lutz B, Marsicano G, Maldonado R, Hillard CJ (2015). The endocannabinoid system in guarding against fear, anxiety and stress. Nat Rev Neurosci.

[13] Steiner MA, Wotjak CT (2008). Role of the endocannabinoid system in regulation of the hypothalamic-pituitary-adrenocortical axis. Prog Brain Res.

[14] Rosales VP, Ikeda K, Hizaki K, Naruo T, Nozoe SI, Ito G (2002). Emotional stress and brux-like activity of the masseter muscle in rats. Eur J Orthod.

[15] Robles EMS, Arias AB, Fontelles MIM (2012). Cannabinoids and muscular pain. Effectiveness of the local administration in rat. Eur J Pain.

[16] Huang F, Zhang M, Chen YJ, Li Q, Wu AZ (2011). Psychological stress induces temporary masticatory muscle mechanical sensitivity in rats. J Biomed Biotechnol.

[17] Denadai-Souza A, Camargo LL, Ribela MTCP, Keeble JE, Costa SKP, Muscará MN (2009). Participation of peripheral tachykinin NK1 receptors in the carrageenan-induced inflammation of the rat temporomandibular joint. Eur J Pain.

[18] Ulrich-Lai YM, Figueiredo HF, Ostrander MM, Choi DC, Engeland WC, Herman JP (2006). Chronic stress induces adrenal hyperplasia and hypertrophy in a subregion-specific manner. Am J Physiol Endocrinol Metab.

[19] Ishikawa M, Hara C, Ohdo S, Ogawa N (1992). Plasma corticosterone response of rats with sociopsychological stress in the communication box. Physiol Behav.

[20] Endo Y, Yamauchi K, Fueta Y, Irie M (2001). Changes of body temperature and plasma corticosterone level in rats during psychological stress induced by the communication box. Med Sci Monit.

[21] Koko V, Djordjeviae J, Cvijiae G, Davidoviae V (2004). Effect of acute heat stress on rat adrenal glands: a morphological and stereological study. J Exp Biol.

[22] Shimizu S, Nakatani Y, Kurose M, Imbe H, Ikeda N, Takagi R (2020). Modulatory effects of repeated psychophysical stress on masseter muscle nociception in the nucleus raphe magnus of rats. J Oral Sci.

[23] Kovács KJ (2008). Measurement of immediate-early gene activation- c-fos and beyond. J Neuroendocrinol.

[24] Mitsikostas DD, del Rio MS (2001). Receptor systems mediating c-fos expression within trigeminal nucleus caudalis in animal models of migraine. Brain Res Brain Res Rev.

[25] Fitzcharles MA, Cohen SP, Clauw DJ, Littlejohn G, Usui C, Häuser W (2021). Nociplastic pain: towards an understanding of prevalent pain conditions. Lancet.

[26] Zieliński G, Pająk-Zielińska B, Ginszt M (2024). A meta-analysis of the global prevalence of temporomandibular disorders. J Clin Med.

[27] Willassen L, Johansson AA, Kvinnsland S, Staniszewski K, Berge T, Rosén A (2020). Catastrophizing has a better prediction for TMD than other psychometric and experimental pain variables. Pain Res Manag.

[28] Ohrbach R, Michelotti A (2018). The role of stress in the etiology of oral parafunction and myofascial pain. Oral Maxillofac Surg Clin North Am.

[29] Jacob MTRJ, Milani BJ (2023). Retrograde inhibition of hyperactive central pathways in nociplastic pain. Braz J Pain.

[30] Russo EB (2016). Clinical endocannabinoid deficiency reconsidered: current research supports the theory in migraine, fibromyalgia, irritable bowel, and other treatment-resistant syndromes. Cannabis Cannabinoid Res.

[31] Rudenko V, Rafiuddin A, Leheste JR, Friedman LK (2012). Inverse relationship of cannabimimetic (R+)WIN 55, 212 on behavior and seizure threshold during the juvenile period. Pharmacol Biochem Behav.

[32] Pascual D, Goicoechea C, Suardíaz M, Martín MI (2005). A cannabinoid agonist, WIN 55,212-2, reduces neuropathic nociception induced by paclitaxel in rats. Pain.

[33] Bluett RJ, Báldi R, Haymer A, Gaulden AD, Hartley ND, Parrish WP (2017). Endocannabinoid signalling modulates susceptibility to traumatic stress exposure. Nat Commun.

[34] Zoppi S, Pérez Nievas BG, Madrigal JLM, Manzanares J, Leza JC, García-Bueno B (2011). Regulatory role of cannabinoid receptor 1 in stress-induced excitotoxicity and neuroinflammation. Neuropsychopharmacology.

[35] McLaughlin RJ, Hill MN, Dang SS, Wainwright SR, Galea LAM, Hillard CJ (2013). Upregulation of CB1 receptor binding in the ventromedial prefrontal cortex promotes proactive stress-coping strategies following chronic stress exposure. Behav Brain Res.

[36] Hsieh GC, Pai M, Chandran P, Hooker BA, Zhu CZ, Salyers AK (2011). Central and peripheral sites of action for CB 2 receptor mediated analgesic activity in chronic inflammatory and neuropathic pain models in rats. Br J Pharmacol.

[37] Cavuoto P, McAinch AJ, Hatzinikolas G, Janovská A, Game P, Wittert GA (2007). The expression of receptors for endocannabinoids in human and rodent skeletal muscle. Biochem Biophys Res Commun.

[38] Dalle S, Koppo K (2021). Cannabinoid receptor 1 expression is higher in muscle of old vs. young males, and increases upon resistance exercise in older adults. Sci Rep.

[39] Nicot R, Raoul G, Vieira AR, Ferri J, Sciote JJ (2023). ACTN3 genotype influences masseter muscle characteristics and self-reported bruxism. Oral Dis.

[40] Cioffi I, Landino D, Donnarumma V, Castroflorio T, Lobbezoo F, Michelotti A (2017). Frequency of daytime tooth clenching episodes in individuals affected by masticatory muscle pain and pain-free controls during standardized ability tasks. Clin Oral Investig.

