# Supplementary material for: Endocannabinoid system attenuates emotional stress-induced orofacial musculoskeletal pain in rats
Source: Braz J Med Biol Res. 2026 Aug 7;59:e15331. doi: 10.1590/1414-431X2026e15331 (PMC13450814; doi:10.1590/1414-431X2026e15331)

**Figure S1.** Experimental protocol. AM251: selective $CB_1$ receptor antagonist; AM630: selective $CB_2$ cannabinoid antagonist; WIN55,212-2: non-selective cannabinoid receptor agonist; TG: trigeminal ganglion; Sp5C: trigeminal nucleus caudalis.

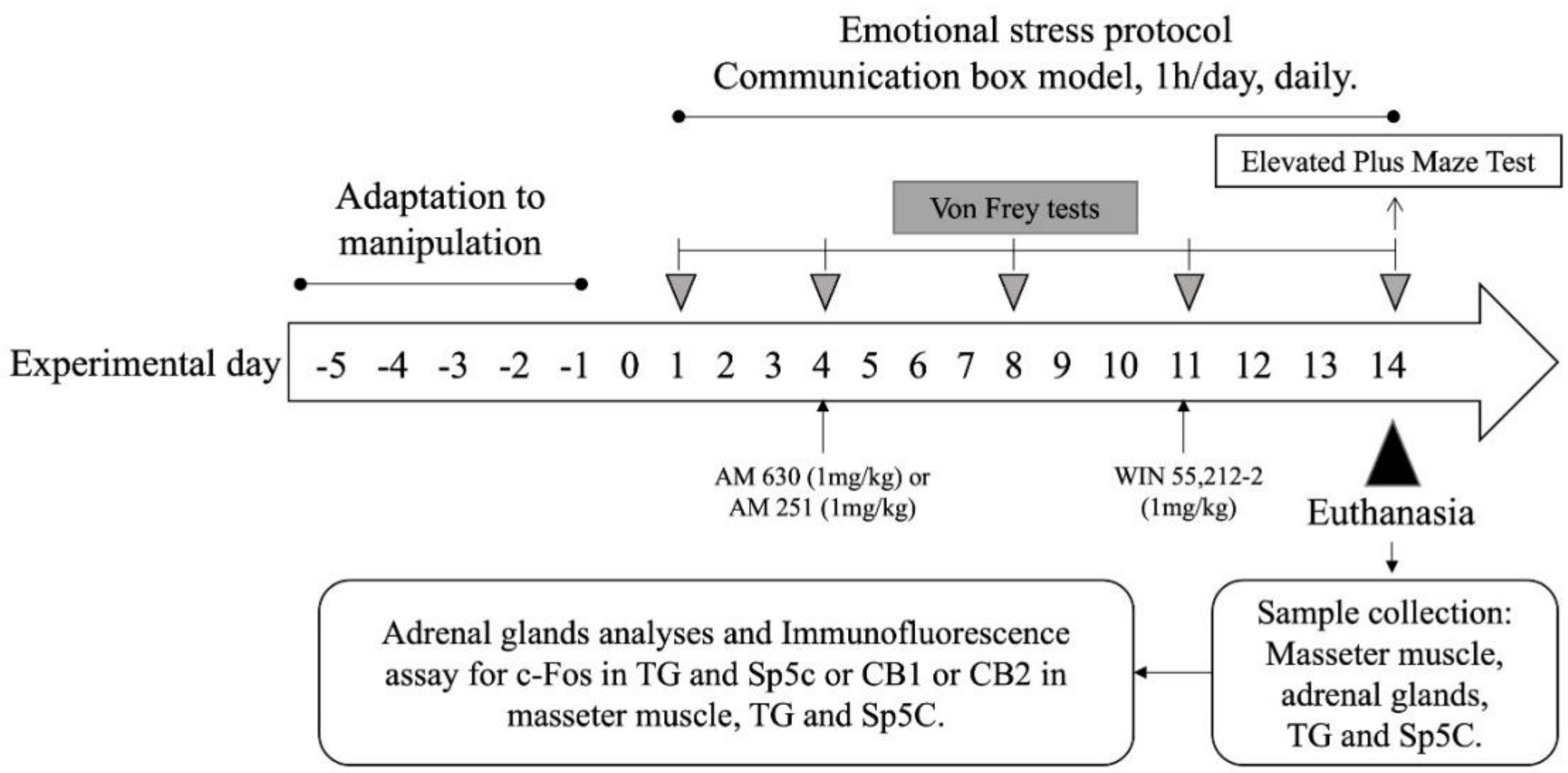

Supplement: Supplementary file 1 [file 1414-431X-bjmbr-59-e15331-suppl.pdf]
